# Variability in oxidative stress-related genes (*SOD2, CAT, GPX1, GSTP1, NOS3, NFE2L2*, and *UCP2*) and susceptibility to migraine clinical phenotypes and features

**DOI:** 10.3389/fneur.2022.1054333

**Published:** 2023-01-09

**Authors:** Maria Papasavva, Michail Vikelis, Vasileios Siokas, Martha-Spyridoula Katsarou, Emmanouil V. Dermitzakis, Athanasios Raptis, Aikaterini Kalliantasi, Efthimios Dardiotis, Nikolaos Drakoulis

**Affiliations:** ^1^Research Group of Clinical Pharmacology and Pharmacogenomics, Faculty of Pharmacy, School of Health Sciences, National and Kapodistrian University of Athens, Athens, Greece; ^2^Headache Clinic, Mediterraneo Hospital, Glyfada, Greece; ^3^Laboratory of Neurogenetics, Department of Neurology, University Hospital of Larissa, Larissa, Greece; ^4^Faculty of Medicine, School of Health Sciences, University of Thessaly, Larissa, Greece; ^5^Euromedica General Clinic, Thessaloniki, Greece

**Keywords:** antioxidant enzymes, single nucleotide polymorphisms (SNPs), primary headaches, migraine genetics, redox homeostasis

## Abstract

**Introduction:**

Migraine is a complex disorder with genetic and environmental inputs. Cumulative evidence implicates oxidative stress (OS) in migraine pathophysiology while genetic variability may influence an individuals' oxidative/antioxidant capacity. Aim of the current study was to investigate the impact of eight common OS-related genetic variants [rs4880 (*SOD2*), rs1001179 (*CAT*), rs1050450 (*GPX1*), rs1695 (*GSTP1*), rs1138272 (*GSTP1*), rs1799983 (*NOS3*), rs6721961 (*NFE2L2*), rs660339 (*UCP2*)] in migraine susceptibility and clinical features in a South-eastern European Caucasian population.

**Methods:**

Genomic DNA samples from 221 unrelated migraineurs and 265 headache-free controls were genotyped for the selected genetic variants using real-time PCR (melting curve analysis).

**Results:**

Although allelic and genotypic frequency distribution analysis did not support an association between migraine susceptibility and the examined variants in the overall population, subgroup analysis indicated significant correlation between *NOS3* rs1799983 and migraine susceptibility in males. Furthermore, significant associations of *CAT* rs1001179 and *GPX1* rs1050450 with disease age-at-onset and migraine attack duration, respectively, were revealed. Lastly, variability in the *CAT, GSTP1* and *UCP2* genes were associated with sleep/weather changes, alcohol consumption and physical exercise, respectively, as migraine triggers.

**Discussion:**

Hence, the current findings possibly indicate an association of OS-related genetic variants with migraine susceptibility and clinical features, further supporting the involvement of OS and genetic susceptibility in migraine.

## 1. Introduction

Migraine is a complex, disabling primary headache disorder with a high worldwide prevalence, estimated ~15%, female preponderance (3:1 female-to-male ratio), and genetic predisposition (1–3). Typically is characterized by recurrent attacks of moderate to severe throbbing headache, lasting 4–72 h, aggravated by routine physical activity and often accompanied by symptoms such as nausea, vomiting, photophobia, and/or phonophobia (4, 5). About 30% of migraine cases undergo transient, reversible focal neurological symptoms, the so-called aura, occurring usually before the headache phase (6, 7). Migraine clinical diagnosis is based on the International Classification of Headache Disorders 3rd Edition (ICHD-III) criteria, which subdivides migraine into two major subtypes with substantial symptomatic overlap, namely migraine without aura (MwoA) and migraine with aura (MwA) (4).

Neurological and vascular mechanisms are believed to be involved in migraine pathophysiology. Main events implicated are cortical spreading depression, activation of the trigeminovascular system, and neurogenic inflammation causing meningeal vasculature changes and the release of various migraine markers. Recent evidence supported an emerging role of metabolic abnormalities, including oxidative stress, in migraine pathogenesis (8, 9). Even though some studies investigating certain markers of oxidative stress are inconsistent, cumulative findings largely indicate an alteration in physiological redox balance in migraine patients characterized by increased oxidative or nitrosative stress and/or reduced antioxidant capacity (10–12). Furthermore, oxidative stress seems to be a common denominator of the most common migraine triggers, which are likely to further enhance oxidative stress levels (13).

Migraine is a multifactorial disease, as most common complex disorders, with a substantial genetic component indicated by family and twin epidemiological studies (14–16). Thus, migraine phenotypes seem to be shaped by genetic susceptibility and exposure to environmental triggers (17, 18). Heritability seems to be more eminent in MwA subtype than MwoA, further supported by the identification of mutations in three ion transporter genes (*CACNA1A, ATP1A2*, and *SCN1A)* associated with familial hemiplegic migraine (FHM); a rare monogenic form of MwA (19). The more common subtypes of migraine are mainly polygenic, with a complex interaction between numerous genetic variants, each having a small genetic effect, conferring disease susceptibility (17, 20). Recent genome-wide association studies (GWAS) identified multiple genetic variants associated with migraine susceptibility (21–26). In addition, genetic factors seem to influence clinical features of common migraine i.e., earlier age of disease onset, increased migraine frequency in males and higher number of days with medication (27).

**Figure 1:**
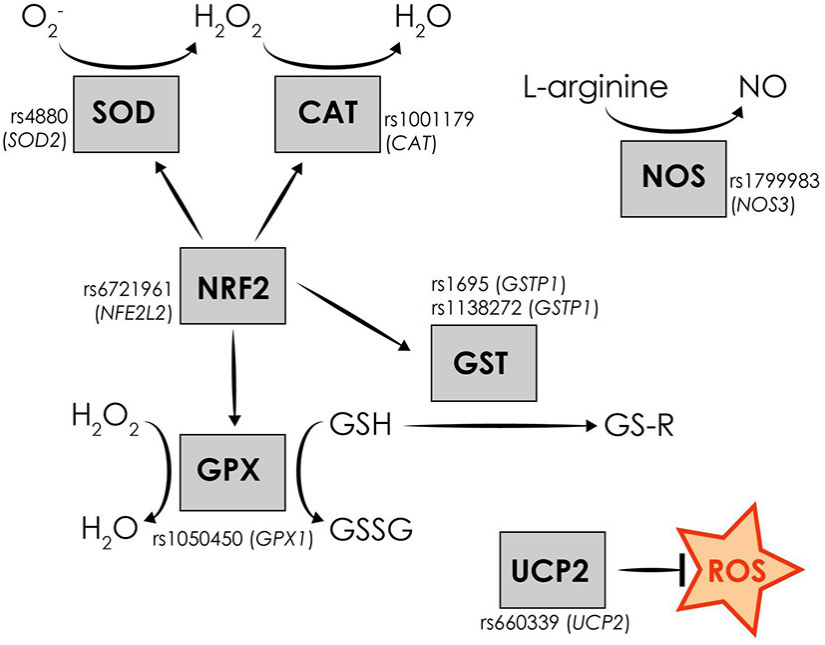
Schematic representation of the investigated oxidative stress-related genetic variants and the function of the encoded oxidative stress-related proteins.

Considering the implication of oxidative stress in migraine pathophysiology and the strong genetic component of the disorder, variation in oxidative stress-related genes may contribute to migraine susceptibility. Single nucleotide polymorphisms (SNPs) in genes encoding for oxidative stress-related proteins may modify protein function resulting in increased oxidative-stress levels associated with various diseases, including migraine. Hence, aim of the current study was to examine the possible association between eight SNPs in oxidative stress-related genes, namely rs4880 (*SOD2*), rs1001179 (*CAT*), rs1050450 (*GPX1*), rs1695 (*GSTP1*), rs1138272 (*GSTP1*), rs1799983 (*NOS3*), rs6721961 (*NFE2L2*), and rs660339 (*UCP2*), and the susceptibility to develop migraine and sub-clinical phenotypes, in South-eastern European Caucasian (SEC) clinically examined patients and controls (Table 1, Figure 1). The frequency distribution of the majority of the investigated SNPs, associated with the incidence of various diseases, was previously examined by Katsarou et al. in a Caucasian population of the Southeastern European region (28). Identifying the genetic factors implicated in the susceptibility to develop migraine clinical phenotypes and features may contribute potentially to discover possible diagnostic biomarkers, to uncover key protein molecules and thus understand more accurately the disease pathophysiology, and ultimately to allow early set-up of treatment and more precise therapeutic strategies.

**Table 1:** Summary of the investigated oxidative stress-related SNPs.

**Gene**	**Locus**	**Protein**	**SNP** **(dbSNP RefSNP)**	**Consequence**	**MAF**		
					**Allele**	**ALL**	**EUR**
*SOD2 - MnSOD*	6q25.3	Superoxide Dismutase 2	rs4880	Missense Variant (p.Val16Ala)	C	0.41	0.47
*CAT*	11p13	Catalase	rs1001179	Upstream Transcript Variant	T	0.13	0.23
*GPX1*	3p21.31	Glutathione Peroxidase 1	rs1050450	Missense Variant (p.Pro200Leu)	A	0.22	0.34
*GSTP1*	11q13.2	Glutathione S-Transferase Pi 1	rs1695	Missense Variant (p.Ile105Val)	G	0.35	0.33
			rs1138272	Missense Variant (p.Ala114Val)	T	0.03	0.07
*NOS3*	7q36.1	Nitric Oxide Synthase 3/Endothelial Nitric Oxide Synthase (eNOS)	rs1799983	Missense Variant (p.Asp298Glu)	T	0.18	0.34
*NFE2L2*	2q31.2	Nuclear Factor, Erythroid 2 Like 2 (NRF2)	rs6721961	Upstream Transcript Variant	T	0.15	0.13
*UCP2*	11q13.4	Uncoupling Protein 2	rs660339	Missense Variant (p.Ala55Val)	A	0.42	0.40

Information retrieved from: https://www.genecards.org/ and https://www.ncbi.nlm.nih.gov/snp/.

SNP, Single Nucleotide Polymorphism; MAF, Minor Allele Frequency; ALL, Global; EUR, European.

## 2. Subjects and methods

### 2.1. Study population

The current case-control study involved 486 unrelated subjects with SEC origin. A total of 221 subjects (37 males and 184 females) diagnosed by experienced neurologists as migraineurs according to the International Classification of Headache Disorders criteria (ICHD-3), aged between 18 and 72 years (mean ± standard deviation: 41.9 ± 11.3 years), served as case group. The case group was prospectively recruited from specialized headache clinics located in Glyfada and Thessaloniki, Greece, between September 2019 and July 2021. The control group consisted of 265 neurologically healthy individuals (133 males, 132 females) with no personal and family history of migraine or any other headache disorder, aged between 21 and 85 years (mean ± standard deviation: 57.7 ± 12.8 years). Control subjects were recruited from the Neurology Department, University Hospital of Larissa, Greece. Data collected from control subjects included only age and gender. Demographic data of the study population, anthropometric data and the main clinical characteristics of migraine cohort are summarized in Table 2. Each study subject was assigned with a unique serial number to conceal their identity. All study subjects signed a written informed consent. Approval was obtained by the appropriate Ethic Committees (Mediterraneo Hospital, Glyfada, Greece, and University Hospital of Larissa) and the research was performed in accordance with the principles outlined in the Declaration of Helsinki.

**Table 2:** Demographic and clinical characteristics of the study population.

	**Migraine patients**		**Headache-free controls**	
	**(*****N*** = **221)**		**(*****N*** = **265)**	
Age (years)^*^	41.9 ± 11.3 ranged from 18 to 72		57.7 ± 12.8 ranged from 21 to 85	
**Gender**, ***n*** **(%)**				
Male	37	(16.7)	133	(50.2)
Female	184	(83.3)	132	(49.8)
**BMI (kg/m** ^**2**^ )^*^	24.6 ± 4.2		–	
**Smoking**, ***n*** **(%)**				
Never	130	(58.8)	–	
Former	27	(12.2)	–	
Ever	64	(29.0)	–	
**Age of diagnosis (years)** ^*^	20.1 ± 8.2 ranged from 5 to 52		–	
**Positive family history**, ***n*** **(%)**	163	(73.8)	–	
**Type of migraine**, ***n*** **(%)**				
1.1. MwoA	127	(57.5)	–	
1.2. MwA	27	(12.2)	–	
1.3. CM	67	(30.3)	–	

* Values are presented as mean ± SD.

BMI, body mass index; MwoA, Migraine without Aura; MwA, Migraine with Aura; CM, Chronic Migraine.

### 2.2. DNA extraction and genotyping

Epithelial cells from the participants oral cavity were collected by sterile buccal swabs. Genomic DNA was extracted from the epithelial cell samples using a commercial nucleic acid isolation kit (Nucleospin Tissue; Macherey-Nagel GmbH & Co., KG, Düren, Germany), according to the manufacturer's instructions. DNA concentration was determined by Nanodrop 2000 Spectrophotometer (Thermo Scientific, USA) and the samples were stored at −20°C until further analysis. Genotyping for the investigated oxidative stress-related SNPs (rs4880-*SOD2*, rs1001179*-CAT*, rs1050450*-GPX1*, rs1695*-GSTP1*, rs1138272-*GSTP1*, rs1799983*-NOS3*, rs6721961*-NFE2L2*, and rs660339*-UCP2*) was carried out by real-time Polymerase Chain Reaction in LightCycler^®^ 480 System (Roche Diagnostics, Germany) using SimpleProbe^®^ probes (LightSNiP assays; TIB Molbiol, Berlin, Germany), followed by melting curve analysis. In particular, DNA samples (50 ng) were amplified using the respective LightSNiP Assay for each SNP and Lightcycler^®^FastStart DNA Master HybProbe Mix (Roche, Germany), according to the following PCR protocol: initial denaturation for 10 min at 95°C, followed by 45 cycles of denaturation for 10 s at 95°C, primer annealing for 10 s at 60°C and extension for 15 s at 72°C, followed by melting curve analysis to determine homozygosity for the wild-type alleles, heterozygosity, and homozygosity for the variant alleles.

### 2.3. Statistical analysis

Continuous data are described as mean ± standard deviation (SD), and categorical data as frequencies (*n*) and percentages (%). Distribution of the continuous variables was examined with Kolmogorov-Smirnov and Shapiro–Wilks tests. The disease age-at-onset (years), current frequency of migraine attacks (days/month) and typical duration of migraine attacks (hours) were not normally distributed, thus non-parametric tests (Mann-Whitney test for two-group comparisons and Kruskal-Wallis test for three-group comparisons) were used to investigate their association with the examined SNPs. Genotype and allele frequencies of the selected SNPs were compared between groups using chi-square (χ^2^; Pearson or Fischer's exact) tests under co-dominant, dominant, recessive, over-dominant genotypic and allelic inheritance models. Contingency 2 × 2 tables were designed and crude odds ratios (OR) with their corresponding 95% confidence intervals (CI) were calculated to examine the association of the investigated SNPs with migraine and migraine subtypes susceptibility, and clinical traits. Logistic regression analysis was also applied to adjust for potential confounding factors including age (continuous), gender (categorical), Body Mass Index (BMI) (continuous), and smoking status (categorical). Two-sided *p-*values < 0.05 were considered statistically significant. However, in some tests the *p*-value threshold automatically reduced to 0.01 to overcome the multiple tests effect, such as the Bonferroni correction etc. Statistical analyses were carried out by the IBM SPSS Statistics software (version 26.0 for Windows), *R* language for statistical computing (violin plots extraction) as well as G^*^Power software for power analysis. Consistency of the genotype frequency distributions with the Hardy–Weinberg Equilibrium (HWE) was examined with chi-square test using the web-based Online Encyclopedia for Genetic Epidemiology studies software (29). Haplotype analyses were carried out using the SHEsis web-based platform (http://analysis.bio-x.cn/myAnalysis.php) (30, 31).

## 3. Results

### 3.1. Analysis of association between oxidative stress-related SNPs and migraine susceptibility

Genotype frequencies of the investigated SNPs were consistent with HWE in both case and control groups (*p* > 0.05), except from the *GPX1 rs1050450* which deviated from the HWE in the control group (*p* = 0.010). The observed genotype and allele frequency distribution of the investigated SNPs did not differ significantly between case and control subjects in any of the genetic inheritance model tested (*p* > 0.05) in the overall SEC population of the study. A statistically significant difference was observed for the *NOS3* rs1799983 variant between migraineurs and control male subjects, as reported in Table 3, although no statistically significant difference was observed between cases and controls in the overall SEC population for the rs1799983 (Table 4). After adjustment, the more common G allele of the rs1799983 (83.8 vs. 65.8%) and homozygous GG genotype (73.0 vs. 43.6%) were statistically more prevalent in male migraineurs compared to the male control group [GG vs. GT + TT: OR_adj_ 2.766 (1.060–7.222), *p*_*adj*_ = 0.038; G vs. T: OR 2.687 (1.378–5.240), *p* = 0.003] (Table 3). Hence, homozygosity for the *NOS3* rs1799983 more common G allele seems to be associated with substantially increased migraine susceptibility in male population. Stratified analysis based on migraine subtypes showed no statistically significant differences in allele or genotype frequency distributions of the examined SNPs among MwoA, MwA, and chronic migraine (CM) patients and migraine-free controls in any of the genetic inheritance model tested (data not shown).

**Table 3:** Genotypic and allelic frequency distribution analysis of the *NOS3* rs1799983 variant in male subjects.

***NOS3*** **rs1799983**	♂ **Migraine cases (*****N*** = **37)**		♂ **Controls (*****N*** = **133)**		**OR (95% CI)**	***p***	OR_adj_ (95% CI)^*^	p_adj_^*^
	***n***	**(%)**	***n***	**(%)**				
GG	27	(73.0)	58	(43.6)	1.0 (reference)	–	–	–^i^
GT	8	(21.6)	59	(44.4)	**3.433 (1.441–8.180)**	**0.004**	2.281 (0.807–6.448)	0.120
TT	2	(5.4)	16	(12.0)	3.724 (0.799–17.359)	0.077	5.259 (0.811–34.087)	0.082
GT + TT	10	(27.0)	75	(56.4)	**3.491 (1.565–7.789)**	**0.002**	**2.766 (1.060–7.222)**	**0.038**
TT	2	(5.4)	16	(12.0)	1.0 (reference)	–	–	–^ii^
GT	8	(21.6)	59	(44.4)	0.922 (0.178–4.776)	1.000^**^	0.423 (0.054–3.325)	0.413
GT + GG	35	(94.6)	117	(88.0)	0.418 (0.092–1.906)	0.368^**^	0.231 (0.036–1.505)	0.126
GT	8	(21.6)	59	(44.4)	1.0 (reference)	–	–	–^iii^
TT + GG	29	(78.4)	74	(55.6)	**0.346 (0.147–0.813)**	**0.012**	0.529 (0.194–1.442)	0.213
G	62	(83.8)	175	(65.8)	1.0 (reference)	–	–	–^iv^
T	12	(16.2)	91	(34.2)	**2.687 (1.378–5.240)**	**0.003**	-	-

OR, Odds Ratio; CI, Confidence Interval.

* Adjusted for age.

** Two-tailed Fisher's Exact test.

i Power = 0.839.

ii Power = 0.882.

iii Power = 0.933.

iv Power = 0.933.

Bold values indicate statistical significance.

**Table 4:** Genotypic and allelic frequency distribution analysis of the *NOS3* rs1799983 variant between migraine cases and control subjects.

***NOS3*** **rs1799983**	**OR (95% CI)**	***P*** **-value**	OR (95% CI)^*^	***P*** -value^*^
**Migraine cases vs. controls**				
GG vs. TT	1.396 (0.748–2.605)	0.294	1.618 (0.711–3.681)	0.252
GG vs. GT	1.188 (0.815–1.732)	0.371	1.112 (0.686–1.803)	0.666
GT vs. TT	1.175 (0.628–2.199)	0.614	1.482 (0.610–3.603)	0.385
GG vs. GT + TT	1.224 (0.855–1.752)	0.269	1.202 (0.756–1.913)	0.436
TT vs. GT + GG	0.779 (0.429–1.415)	0.412	0.632 (0.282–1.415)	0.264
GT vs. GG + TT	0.894 (0.624–1.282)	0.543	0.970 (0.609–1.545)	0.898
G vs. T	1.182 (0.901–1.550)	0.226	-	-

* Adjusted for age and gender.

### 3.2. Analysis of association between oxidative stress-related SNPs and migraine clinical features

**Figure 2:**
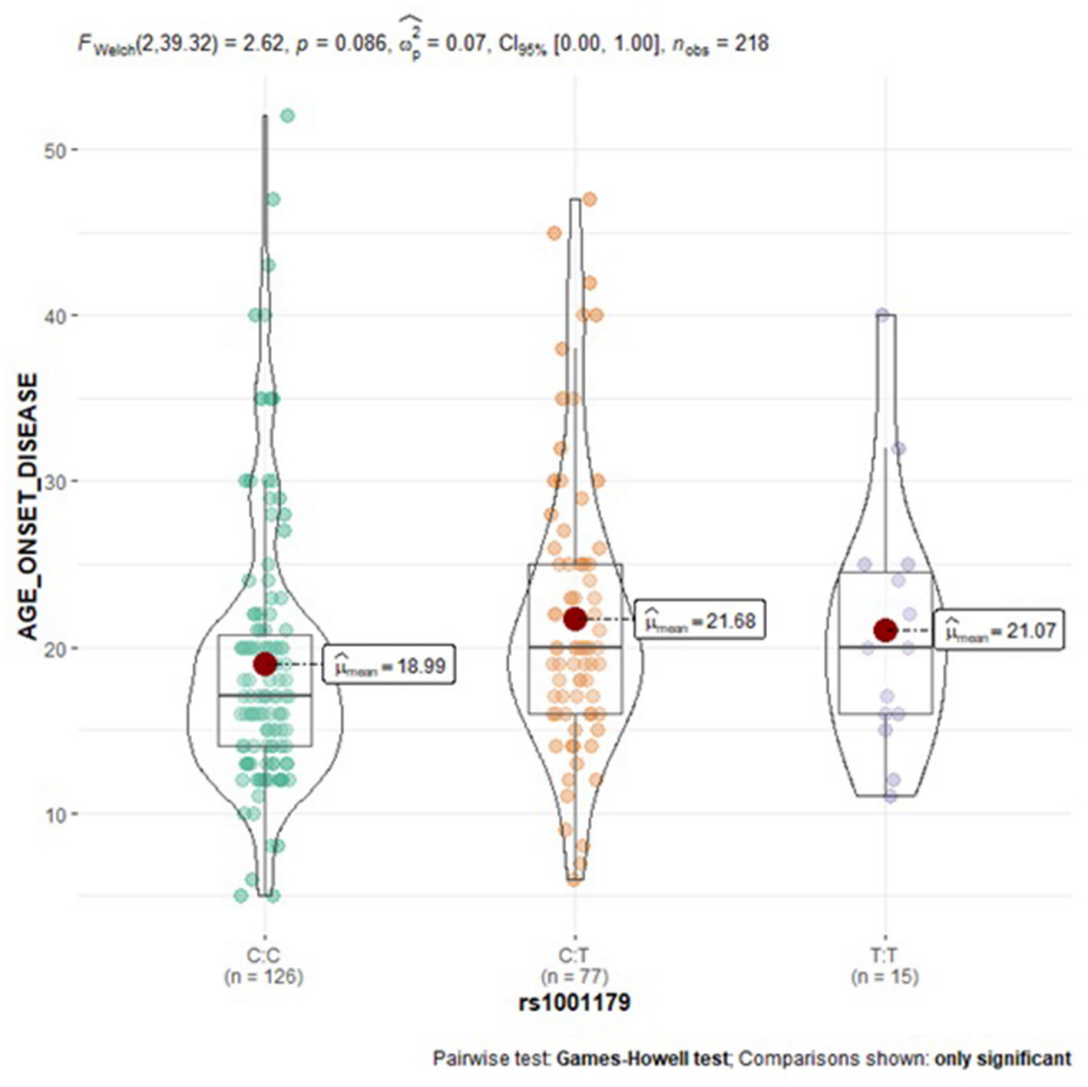
Violin plots displaying the mean age at disease onset according to *CAT* rs1001179 genotype profile in migraine subjects.

Subgroup analysis of the examined SNPs and disease clinical features (age at onset, attack frequency, and attack duration) in migraine group was performed to assess genotype-phenotype associations. As reported in Table 5, a statistically significant trend of association was revealed for the *CAT* rs1001179 variant with the disease age at onset. In particular, homozygosity for the minor T allele and heterozygosity were associated with a later age at onset (CT: 21.68 ± 8.40-years-old; TT: 21.07 ± 7.60-years-old) as compared to the homozygosity for the more common C allele (CC: 18.99 ± 8.06-year-old); consequently, the rs1001179 variant T allele may serve as a genetic factor possibly leading to a later age at onset of migraine (Figure 2). Moreover, a statistically significant association between *GPX1* rs1050450 variant and migraine attack duration was observed. The rs1050450 variant T allele (40.0 vs. 27.8%) and TT homozygosity (16.4 vs. 7.0%) were significantly more prevalent in patients with longer attack duration (>24 h) as compared to patients with shorter attack duration (≤ 24 h) [C vs. T: OR 1.727 (1.097–2.719), *p* = 0.018; TT vs. CT + CC: OR_adj_ 0,362 (0.138–0.951), *p*_*adj*_ = 0.039] (Table 6). In addition, a trend of association was indicated between *NOS3* rs1799983 variant T allele and longer attack duration (>24 h); homozygous and heterozygous carriers of the rs1050450 variant T allele (62.5 vs. 37.5%) seem to experience migraine attacks longer than 24 h as compared to homozygous for the more common G allele [G vs. T: OR 1.481 (0.941–2.332), *p* = 0.089; GG vs. GT + TT: OR_adj_ 1.731 (0.910–3.291), *p*_*adj*_ = 0.094] (Table 7). No statistically significant association was indicated for the *SOD2* rs4880, *GSTP1* rs1695, and rs1138272, *NFE2L2* rs6721961, and *UCP2* rs660339 variants with age at onset, attack frequency and attack duration in the SEC migraine subjects of the current study.

**Table 5:** Analysis of the *CAT* rs1001179 variant association with clinical features in migraineurs.

***CAT rs1001179***	**CC**	**CT**	**TT**	***p*** ^*^	**CT+TT**	***p*** ^a^ ^**^	**CC+CT**	***p*** ^b^ ^**^	**CC+TT**	***p*** ^c^ ^**^
Migraineurs subjects (*N* = 218)	126	77	15		92		203		141	
Age at onset	18.99 ± 8.06	21.68 ± 8.40	21.07 ± 7.60	**0.010**^d^, ^i^	21.58 ± 8.24	**0.002** ^ii^	20.01 ± 8.27	0.435	19.21 ± 8.01	**0.006** ^iii^
(years)	17 (5–52)	20 (6–47)	20 (11–40)		20 (6–47)		19 (5–52)		17 (5–52)	
Attack frequency	10.66 ± 8.26	11.56 ± 9.22	10.73 ± 7.06	0.859	11.43 ± 8.88	0.605	11.00 ± 8.63	0.729	10.67 ± 8.12	0.726
(days/month)	8 (0.25–30)	10 (0.33–30)	10 (2–25)		10 (0.33–30)		9 (0.25–30)		8 (0.25–30)	

Data are presented as mean ± SD and median (min-max). Bold values are considered statistically significant (*p* < 0.05).

* Kruskal-Wallis Test.

** Mann-Whitney Test.

a CT + TT vs. CC.

b CC + CT vs. TT.

c CC + TT vs. CT.

d CC vs. CT adjusted by the Bonferroni correction for multiple tests *p* = 0.010.

i Power = 0.917.

ii Power = 0.922.

iii Power = 0.998.

**Table 6:** Genotypic and allelic frequency distribution analysis of the *GPX1* rs1050450 variant in migraineurs according to the typical duration of migraine attacks (≤24 vs. >24 h).

***GPX1*** **rs1050450**	**Attack duration (*****N*** = **213)**				**OR (95% CI)**	***p***	OR_adj_ (95% CI)^*^	***p_adj_*** ^*^
	≤**24 h (*****N*** = **158)**		>**24 h (*****N*** = **55)**					
	***N***	**(%)**	***n***	**(%)**				
CC	81	(51.3)	20	(36.4)	1.0 (reference)	–	–	–^i^
CT	66	(41.8)	26	(47.3)	1.595 (0.819–3.110)	0.168	1.559 (0.796–3.057)	0.196
TT	11	(7.0)	9	(16.4)	**3.314 (1.210–9.077)**	**0.023** ^**^	**3.973 (1.345–11.734)**	**0.013**
CT + TT	77	(48.7)	35	(63.6)	1.841 (0.979–3.463)	0.057	1.784 (0.943–3.375)	0.075
CT	66	(41.8)	26	(47.3)	1.0 (reference)	–	–	–^ii^
TT	11	(7.0)	9	(16.4)	2.077 (0.771–5.595)	0.143	2.223 (0.793–6.232)	0.129
TT + CC	92	(58.2)	29	(52.7)	0.800 (0.432–1.482)	0.478	0.836 (0.448–1.560)	0.573
TT	11	(7.0)	9	(16.4)	1.0 (reference)	–	–	–^iii^
CT + CC	147	(93.0)	46	(83.6)	**0.382 (0.149–0.980)**	**0.040**	**0.362 (0.138–0.951)**	**0.039**
C	228	(72.2)	66	(60.0)	1.0 (reference)	–	–	–^iv^
T	88	(27.8)	44	(40.0)	**1.727 (1.097–2.719)**	**0.018**	-	-

* Adjusted for age, gender, BMI, and smoking status.

** Two-tailed Fisher's Exact test.

Bold values are considered statistically significant (*p* < 0.05).

i Power = 0.617.

ii Power = 0.821.

iii Power = 0.807.

iv Power = 0.901.

**Table 7:** Genotypic and allelic frequency distribution analysis of the *NOS3* rs1799983 variant in migraineurs according to the typical duration of migraine attacks (≤ 24 vs. >24 h).

***NOS3*** **rs1799983**	**Attack duration (*****N*** = **220)**				**OR (95% CI)**	***p***	OR (95% CI)^*^	***p_adj_*** ^*^
	≤ **24 h (*****N*** = **164)**		>**24 h (*****N*** = **56)**					
	***n***	**(%)**	***n***	**(%)**				
GG	86	(52.4)	21	(37.5)	1.0 (reference)	–	–	–^i^
GT	64	(39.0)	29	(51.8)	1.856 (0.971–3.548)	0.060	1.780 (0.906–3.500)	0.094
TT	14	(8.5)	6	(10.7)	1.755 (0.603–5.110)	0.371^**^	1.715 (0.572–5.144)	0.336
GT + TT	78	(47.6)	35	(62.5)	1.838 (0.987–3.422)	0.053	1.731 (0.910–3.291)	0.094
GT	64	(39.0)	29	(51.8)	1.0 (reference)	-	-	-^ii^
TT	14	(8.5)	6	(10.7)	0.946 (0.330–2.709)	0.917	1.054 (0.350–3.175)	0.925
TT + GG	100	(61.0)	27	(48.2)	0.596 (0.323–1.098)	0.095	0.644 (0.341–1.215)	0.174
TT	14	(8.5)	6	(10.7)	1.0 (reference)	–	–	–^iii^
GT + GG	150	(91.5)	50	(89.3)	0.778 (0.284–2.132)	0.625	0.738 (0.263–2.067)	0.563
G	236	(72.0)	71	(63.4)	1.0 (reference)	–	–	–^iv^
T	92	(28.0)	41	(36.6)	1.481 (0.941–2.332)	0.089	–	–

* Adjusted for age, gender, BMI, and smoking status.

** Two-tailed Fisher's Exact test.

i Power = 0.657.

ii Power = 0.814.

iii Power = 0.811.

iv Power = 0.913.

### 3.3. Analysis of association between oxidative stress-related SNPs and migraine triggers

The most reported migraine triggering factors by the case subjects of the current study were stress (71.5%), alcohol (38.5%), sleep changes (33.0%), weather changes (26.2%), water deprivation (dehydration; 25.3%), and physical activity (19.0%). Since migraine triggers seem to be capable of generating oxidative stress, an association analysis of the investigated oxidative stress-related SNPs and migraine triggers was performed. Statistically significant associations were indicated for the *CAT* rs1001179 genetic variant with sleep [CT vs. CC + TT: OR_adj_ 0.427 (0.222–0.821), *p*_*adj*_ = 0.011] and weather [TT vs. CT + CC: OR_adj_ 3.164 (1.022–9.801), *p*_*adj*_ = 0.046] changes (Table 8); for the *GSTP1* Val105/Val114 or “G_rs1695_T _rs1138272_” haplotype (*GSTP*1^*^C) with alcohol consumption [OR 2.929 (1.210–7.094), *p* = 0.013] (Table 9); and for the *UCP2* rs660339 genetic variant with physical activity as triggering factor for migraine attacks [CC vs. CT + TT: OR_adj_ 2.135 (1.046–4.358), *p*_*adj*_ = 0.037; C vs. T: OR 1.697 (0.994–2.899), *p* = 0.051; Table 10].

**Table 8:** Association analysis of the *CAT* rs1001179 variant with migraine triggers.

**Sleep changes**	**GG**	**(%)**	**GA**	**(%)**	**AA**	**(%)**		**OR (95% CI)**	***p***	OR_adj_ (95% CI)^*^	***p_adj_*** ^*^
Yes (*n* = 71)	46	(64.8)	17	(23.9)	8	(11.3)	GG vs. **GA vs. AA**	-	**0.013**	-	**0.017** ^i^
No (*n* = 131)	69	(52.7)	56	(42.7)	6	(4.6)	GG vs. GA + AA	1.653 (0.911–2.999)	0.097	1.604 (0.878–2.931)	0.125
	**G**	**(%)**	**A**	**(%)**			AA vs. GA + GG	2.646 (0.880–7.955)	0.087^**^	2.816 (0.923–8.593)	0.069
Yes (*n* = 142)	109	(76.8)	33	(23.2)			GA vs. GG + AA	**0.422 (0.221–0.804)**	**0.008**	**0.427 (0.222–0.821)**	**0.011** ^ii^
No (*n* = 262)	194	(74.0)	68	(26.0)			G vs. A	1.158 (0.718–1.866)	0.547	-	-
**Weather changes**	**GG**	**(%)**	**GA**	**(%)**	**AA**	**(%)**					
Yes (*n* = 57)	32	(56.1)	18	(31.6)	7	(12.3)	GG vs. **GA vs. AA**		0.154		0.117^iii^
No (*n* = 145)	83	(57.2)	55	(37.9)	7	(4.8)	GG vs. GA + AA	0.956 (0.515–1.774)	0.887	0.964 (0.510–1.822)	0.910
	**G**	**(%)**	**A**	**(%)**			AA vs. GA + GG	2.760 (0.922–8.262)	0.071^**^	**3.164 (1.022–9.801)**	**0.046** ^iv^
Yes (*n* = 114)	82	(71.9)	32	(28.1)			GA vs. GG + AA	0.755 (0.394–1.449)	0.398	0.717 (0.366–1.403)	0.331
No (*n* = 290)	221	(76.2)	69	(23.8)			G vs. A	0.800 (0.490–1.306)	0.372	-	-

* Adjusted for age, gender, BMI, and smoking status.

** Two-tailed Fisher's Exact test.

Bold values are considered statistically significant (*p* < 0.05).

i Power = 0.975.

ii Power = 0.999.

iii Power = 0.975.

iv Power = 0.999.

**Table 9:** Haplotype association analysis of the *GSTP1* rs1138272 and rs1695 variants with alcohol as migraine trigger.

			**Alcohol consumption**				**OR (95% CI)**	***p***
	**rs1695**	**rs1138272**	**Yes (%)**		**No (%)**			
H1	A	C	105.00	(69.1)	169.00	(76.1)	0.701 (0.441–1.112)	0.130
H2	G	C	32.00	(21.1)	45.00	(20.3)	1.049 (0.630–1.745)	0.854
H3	G	T	15.00	(9.9)	8.00	(3.6)	2.929 (1.210–7.094)	**0.013** ^i^

Bold values are considered statistically significant (*p* < 0.05).

i Power = 0.820.

**Table 10:** Association analysis of the *UCP2* rs660339 variant with physical activity as migraine trigger.

**Physical activity**	**CC**	**(%)**	**CT**	**(%)**	**TT**	**(%)**		**OR (95% CI)**	***p***	OR_adj_ (95% CI)^*^	***p_adj_*** ^*^
Yes (*n* = 39)	20	(51.3)	15	(38.5)	4	(10.3)	CC vs. CT vs. TT	-	0.110	-	0.111^i^
No (*n* = 159)	53	(33.3)	80	(50.3)	26	(16.4)	CC vs. CT + TT	**2.105 (1.036–4.279)**	**0.037**	**2.135 (1.046–4.358)**	**0.037**
	**C**	**(%)**	**T**	**(%)**			TT vs. CT + CC	0.585 (0.191–1.786)	0.341	0.588 (0.189–1.826)	0.358
Yes (*n* = 78)	55	(70.5)	23	(29.5)			CT vs. CC + TT	0.617 (0.302–1.263)	0.184	0.599 (0.290–1.237)	0.166^ii^
No (*n* = 318)	186	(58.5)	132	(41.5)			C vs. T	1.697 (0.994–2.899)	0.051	-	-

* Adjusted for age, gender, BMI, and smoking status.

Bold values are considered statistically significant (*p* < 0.05).

i Power = 0.441.

ii Power = 0.709.

## 4. Discussion

Cumulative evidence points toward migraine as a conserved adaptive response that ameliorates detrimental oxidative stress and rebalances energy homeostasis in the brain, associated with reproductive or survival advantages as signified by its high prevalence and its correlation with common genetic polymorphisms (11, 32). Besides several biochemical studies revealing diverse metabolic abnormalities in migraineurs, genetic studies assist the hypothesis that migraine patients show an increased vulnerability to oxidative stress, impaired mitochondrial functioning and/or metabolic derangements (11, 12). In addition, migraine often appears as a symptom to other oxidative stress associated concomitant diseases, such as brain tumors and fibromyalgia, further supporting an association between migraine and redox imbalance (33–36). Furthermore, oxidative stress seems to be a common metabolic denominator for the most frequently reported migraine triggers (13). Genetic susceptibility can contribute to decreased antioxidant capacity or increased oxidative stress (37). In the current study, it was hypothesized that an “oxidation predisposed” genetic make-up according to the investigated single nucleotide variants may be associated with the susceptibility to develop migraine and/or diverse clinical phenotypes and features. Thus, the current study analyzed the genotypic and allelic frequency distribution of eight genetic variants in oxidative-stress related proteins (rs4880-*SOD2*, rs1001179*-CAT*, rs1050450*-GPX1*, rs1695*-GSTP1*, rs1138272-*GSTP1*, rs1799983*-NOS3*, rs6721961*-NFE2L2*, and rs660339*-UCP2*) in clinically confirmed migraine subjects and a headache-free control group with SEC origin, in order to investigate their association with migraine susceptibility and diverse clinical phenotypes.

**Figure 3:**
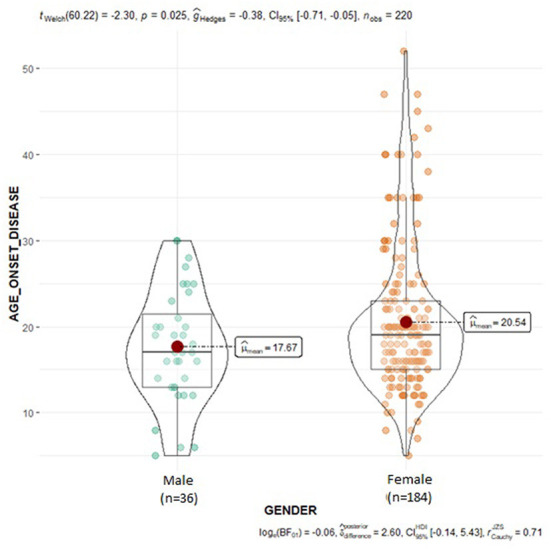
Violin plots displaying the mean age at disease onset in male and female migraine subjects.

The *NOS3* gene, encoding for endothelial nitric oxide synthase (eNOS) enzyme, is mapped on chromosome 7 (7q35–36) and consists of 26 exons (38). The rs1799983 missense variant in exon 7 of the *NOS3* gene is a guanine (G) to thymine (T) replacement (G894T), resulting in the amino acid substitution Glu298Asp (39). This genetic variant may modify eNOS function and has been associated by some studies with decreased NO levels in carriers of the variant T (298Asp) allele (40–42). Borroni et al. suggested that eNOS Asp298 homozygosity is an independent risk factor for MwA (43), while subsequent studies found no association between rs1799983 variation and migraine susceptibility (37, 44–49). In addition, Eroz et al. observed that heterozygosity (GT) and homozygosity for the variant T allele (TT) were significantly more prevalent in migraineurs compared to control subjects (50). Although accordingly with most prior studies, no significant association between migraine susceptibility and rs1799983 variant was observed in the overall SEC population of the current study, subgroup analysis demonstrated that homozygosity for the more common G allele of the rs1799983 variant in the *NOS3* gene seems to be associated with remarkably increased migraine susceptibility in the male population of the current study; thus, *NOS3* rs1799983 may serve as an independent risk factor for migraine susceptibility in male population. Besides diverse ethnical background, the observed inconsistency of the findings concerning the association of the rs1799983 with migraine susceptibility may be attributable to the greater percentage of female participants in most studies due to the female preponderance of migraine; consequently, the results largely reflect the association of the *NOS3* genetic variant with migraine in female population. Genetic influence seems to be more robust in male migraineurs, as suggested by findings from GWAS in migraine, probably due to the considerable environmental and hormonal effect on disease prevalence in females (27). The aforementioned data in addition to the evidence for an association between stronger migraine family history and lower age-at-onset (27), could also possibly explain the lower age-at-onset observed in male patients of the current study compared to female migraineurs (Figure 3). Furthermore, in the overall migraine population, the *NOS3* rs1799983 variant T allele showed a trend of association with migraine attack duration longer than 24 h, with homozygous and heterozygous carriers of the variant T allele experiencing longer migraine attacks as compared to homozygous for the more common G allele. Likewise, in a study by Güler et al., the rs1799983 TT genotype was significantly more prevalent in patients with headache duration >24 h compared to patients with duration <24 h (51). Contrarily, no significant association of the *NOS3* rs1799983 variant with attack duration was observed in a study by Eröz et al. (50).

The gene encoding for catalase (CAT), a crucial endogenous antioxidant enzyme which detoxifies hydrogen peroxide (H_2_O_2_), is located on chromosome 11 (11p13) (52). The rs1001179 (C262T) variant in the promoter region of the *CAT* gene causes alteration at the transcription factor binding site (53, 54). The variant T allele seems to confer enhanced transcriptional rate, while data concerning the influence of the rs1001179 on enzyme activity are controversial (55). Saygi et al. reported no significant differences in *CAT* rs1001179 genotype or allele frequency distribution among children and adolescents with MwA, MwoA, and controls (56). Similarly, Gentile et al. found no significant differences in genotype and allele frequencies between female chronic migraine population and healthy controls (37). Although not correlated to migraine and migraine subtypes *per se*, accordingly with previous studies, the rs1001179 variant in the *CAT* gene seems to delay disease onset in the migraine population of the current study; TT and CT genotypes were associated with a later age at onset, feasibly indicating a delayed migraine onset for the carriers of the variant T allele. In addition, homozygosity for the variant T allele of the *CAT* rs1001179 seems to be associated with sleep and weather changes as triggering factors inducing migraine attacks. Epidemiological data showed a correlation between early migraine onset and enhanced relative risk in first-degree relatives, indicating a genetic component in migraine onset (27). Therefore, the findings of the current study point toward *CAT* as a candidate disease modifying genetic factor.

The enzyme glutathione peroxidase 1 (GPX1) is a major endogenous selenium-dependent antioxidant in defense against oxidative stress. The human *GPX1* gene encoding for glutathione peroxidase is located on chromosome 3 (3p21) and contains the non-synonymous rs1050450 variant which is a C to T alteration in exon 2, resulting in an amino acid substitution from proline (Pro) to leucine (Leu) (57, 58). This genetic variant is associated with altered enzyme activity and may potentially influence a person's antioxidant capacity. In particular, the minor T (Leu) allele has been associated with decreased GPx1 activity (54, 59–61). To the author's knowledge, this is the first study investigating the relationship of the *GPX1* rs1050450 variant and migraine phenotypes. The results of the current study indicated an association between *GPX1* rs1050450 variant and migraine attack duration. In particular, a significantly more frequent prevalence of the variant T allele and TT homozygosity was observed in patients with longer attack duration (>24 h) compared to patients with shorter attack duration (≤24 h); thus, the presence of the variant T allele seems to be related with prolonged migraine attacks in the SEC migraine population of the study. A study by Alp et al. indicated a significant negative correlation between total thiol (-SH) levels and headache duration in patients with MwoA (62). Considering the correlation of the variant T allele with reduced GPX1 activity and the negative correlation of the total -SH levels with migraine attack duration, carriers of the T allele may experience longer attack duration due to reduced antioxidant capacity and thus reduced ability to neutralize oxidants possibly leading in prolonged migraine attacks.

Glutathione S-transferases (GSTs) constitutes a family of phase II xenobiotic metabolizing enzymes that catalyze the conjugation of reduced glutathione (GSH) with hydrophobic electrophilic compounds to generate readily excretable or less toxic metabolites; thus, they can detoxify various harmful substances including reactive oxygen species (ROS) (63, 64). Several factors can influence the activity of this antioxidant enzyme including polymorphic genetic variants (65). The *GSTP1* gene is located on chromosome 11 (11q13) (66). The most reported *GSTP1* genetic variants are the rs1695; an A to G transition at nucleotide 313 resulting in an isoleucine (Ile) to valine (Val) substitution (I105V) in exon 5, and the rs1138272; a G to T transition at nucleotide 341 resulting in an alanine (Ala) to valine (Val) (A114V) substitution in exon 6. These genetic variants result in decreased enzyme activity and detoxification capacity of the protein (67, 68). The current study did not detect any significant association of the *GSTP1* variants with the susceptibility to develop migraine or migraine subtypes and clinical phenotypes. Likewise, Gentile et al. found no significant association of the rs1645 variant with CM susceptibility (37). Nevertheless, a significant association of alcohol consumption reported as migraine trigger with the presence of the variant allele haplotype of *GSTP1* rs1695 and rs1138272 [Val105/Val114 or G_rs1695_T _rs1138272_ (*GSTP*1^*^C) haplotype] was revealed in the migraine cohort of the current study. Alcohol consumption can increase brain oxidative stress through various mechanisms, mainly due to its metabolism by CYP2E1 enzyme and the subsequent production of ROS (69). The isoenzyme Glutathione S-Transferase Pi 1 (GSTP1) can promote brain detoxification and cell protection by modifying the effect of neurotoxins and OS products (70). The Val105/Val114 haplotype is associated with lower enzymatic activity leading to incomplete catabolization of toxicants and potentially to higher oxidative stress levels (67). Therefore, the investigated functional genetic variants, which result in the substitution of two amino acids in the enzyme active site altering its activity and therefore its antioxidant function (71), may render the brain more vulnerable to oxidative damage reinforced by alcohol consumption.

Mitochondrial uncoupling protein 2 (UCP2) is an anion transporter located in the inner mitochondrial membrane (72). UCP2 is widely expressed, including immune system and subcortical brain structures, and is involved in oxidative stress, cellular homeostasis, energy production, and cell survival. A major function of UCP2 is dissipating proton gradient energy and suppressing the generation of ROS (73, 74). UCP2 downregulation is associated with enhanced oxidative stress and inflammation (75). Hence, UCP2 acts protectively against cell death induced by ROS in the central nervous system (76). The *UCP2* gene is located on chromosome 11 (11q13) (77). One of the most reported *UCP2* variant is the missense variant rs660339 in exon 4, a C to T transition, resulting in an amino acid substitution at position 55 of the UCP2 (Ala55Val) which seems to modify the uncoupling degree and consequently protein activity (72). In particular, the Val/Val genotype has been associated with lower degree of uncoupling, increased metabolic efficiency, lesser fat oxidation, reduced energy expenditure, higher exercise efficiency, higher risk of obesity and diabetes, elevated atherogenic index, and greater weight loss compared to the Ala/Val and Ala/Ala genotypes (77, 78). UCP2 demonstrates tissue specific physiological effects as suggested by its tissue-specific regulation e.g., in the brain UCP2 functions as a regulator of oxidative stress. Therefore, the effects of *UCP2* variants may be tissue depended (77). The results of the current study indicated no significant association of the *UCP2* rs660339 variant with migraine susceptibility and clinical phenotypes. However, a significant association was revealed with physical activity as triggering factor for migraine attacks; CC (Ala/Ala) genotype and C (Ala) allele were significantly more prevalent among migraineurs reporting physical activity as migraine trigger. Ahmetov et al. suggested an association between the variant T (Val) allele and higher maximum oxygen uptake (VO_2max_) (79, 80). Additionally, a large cross-sectional population-based study by Hagen et al. revealed an inverse correlation between peak oxygen uptake (VO_2peak_) and migraine in adults aged between 20 and 50 years, with significantly enhancing prevalence of migraine with lower VO_2peak_. Furthermore, a strong association of physical activity with migraine aggravation was observed in adult subjects younger than 50 years-old in the lowest VO_2peak_ quantile. Consequently, the authors suggested that the inverse relationship between headache and VO_2peak_ may be elucidated by shared genetic predisposition factors between migraine and low VO_2peak_ status (81). Taken together, the findings of the current study may indicate *UCP2* gene as a candidate genetic factor predisposing to both migraine and low VO_2peak_, with homozygous patients for the *UCP2* rs660339 wild-type C (Ala) allele reporting more frequently intense physical activity as migraine trigger possibly due to lower VO_2peak_ levels. To the authors' knowledge, this is the first report investigating the association of the *UCP2* rs660339 variant with migraine phenotypes and features. Further larger-scale studies estimating VO_2max_ levels alongside the identification of the genotypic profile for the rs660339 variant and possibly additional variants in the *UCP2* gene are needed to validate the findings of the current study.

Certain limitations of the current study should be acknowledged. Firstly, the study population was limited to SEC migraineurs and headache-free controls to avoid biased introduced by genetic variability within different populations, possibly rendering the findings relevant only for this specific population. Secondly, the sample size was relatively small, particularly in migraine subgroups, thus the effect of low frequency alleles might not be detected. Moreover, the functional implication of the investigated genetic variants on the encoded antioxidant proteins and other related biomarkers was not examined, restricting the acquisition of additional information. Finally, the current study examined only a limited number of oxidative stress-related SNPs. Since migraine is a multifactorial disease influenced by multiple genes, the combined role of the examined genes and other functional genes and loci in migraine susceptibility and clinical phenotypes in SEC and other populations needs to be further investigated.

## 5. Conclusion

In conclusion, the study provides supportive evidence for potential implication of OS-related SNPs in migraine susceptibility and associated clinical phenotypes and features, i.e., age-at-onset, attack duration, and migraine triggers, in a SEC case-control population. Migraine is a common multifactorial disorder with several small effect size genetic variants, together with environmental factors conferring disease susceptibility. Unraveling the genetic susceptibility of migraine phenotypes and features could potentially contribute to disease diagnosis, to more accurate understanding of disease pathophysiology, and eventually to the identification of novel targets for therapeutic treatment. An enigma although remains the magnitude of genetic susceptibility for migraine phenotypes and clinical features and if this is relevant for both genders to the same degree. While oxidative stress and genetic variability seem to play a key role in the pathophysiology of migraine, the precise link between these factors has not been fully understood yet. The current study examines for the first time the potential association of multiple common oxidative-stress related genetic variants with migraine susceptibility and clinical phenotypes in a SEC population. The possible association of certain OS-related genetic variants with migraine features indicated by the current study further supports the involvement of OS-related mechanisms in migraine pathophysiology. Nonetheless, larger-scale multicenter studies are needed to extend and further validate the current findings in SEC and other populations, considering gene-gene and gene environment interactions. The current findings could potentially assist in migraineurs risk stratification strategies and contribute to precision diagnosis and therapy of migraine.

## Data availability statement

The raw data supporting the conclusions of this article will be made available by the authors, without undue reservation.

## Ethics statement

The studies involving human participants were reviewed and approved by Ethics Committee of Mediterraneo Hospital, Glyfada, Greece and University Hospital of Larissa Ethics Committee. The patients/participants provided their written informed consent to participate in this study.

## Author contributions

MP conceptualized the study, performed the research and data analysis, and wrote the manuscript. MP and M-SK designed the experiments. ND supervised the study. MV, VS, EVD, and ED contributed to clinical data and specimen collection. MP and AK analyzed the specimens. ND, VS, and ED revised the study. AR revised the data analysis. All authors have read and approved the final manuscript.
